# A cell based high-throughput screening approach for the discovery of new inhibitors of respiratory syncytial virus

**DOI:** 10.1186/1743-422X-10-19

**Published:** 2013-01-10

**Authors:** Dong-Hoon Chung, Blake P Moore, Daljit S Matharu, Jennifer E Golden, Clinton Maddox, Lynn Rasmussen, Melinda I Sosa, Subramaniam Ananthan, E Lucile White, Fuli Jia, Colleen B Jonsson, William E Severson

**Affiliations:** 1 Current address: Center for Predictive Medicine for Biodefense and Emerging Infectious Diseases, University of Louisville, Louisville, KY, USA; 2 Current Address: Department of Microbiology and Immunology, University of Louisville, Louisville, KY, USA; 3 Southern Research Specialized Biocontainment Screening Center, Southern Research Institute, Birmingham, AL, USA; 4 University of Kansas Specialized Chemistry Center, University of Kansas, Lawrence, KS, USA; 5 Current Address: Department of Biochemistry & Molecular Genetics, University of Alabama at Birmingham, Birmingham, AL, USA; 6 Current Address: Department of Molecular & Cellular Biology, Baylor College of Medicine, Houston, TX, USA

**Keywords:** Aryl sulfonylpyrrolidines, Anti-infective drugs, Automation/robotics, Cell-based assays, HTS, Human respiratory syncytial virus (hRSV), Sulfonamides

## Abstract

**Background:**

Human respiratory syncytial virus (hRSV) is a highly contagious pathogen and is the most common cause of bronchiolitis and pneumonia for infants and children under one year of age. Worldwide, greater than 33 million children under five years of age are affected by hRSV resulting in three million hospitalizations and 200,000 deaths. However, severe lower respiratory tract disease may occur at any age, especially among the elderly or those with compromised cardiac, pulmonary, or immune systems. There is no vaccine commercially available. Existing therapies for the acute infection are ribavirin and the prophylactic humanized monoclonal antibody (Synagis® from MedImmune) that is limited to use in high risk pediatric patients. Thus, the discovery of new inhibitors for hRSV would be clinically beneficial.

**Results:**

We have developed and validated a 384-well cell-based, high-throughput assay that measures the cytopathic effect of hRSV (strain Long) in HEp-2 cells using a luminescent-based detection system for signal endpoint (Cell Titer Glo®). The assay is sensitive and robust, with Z factors greater than 0.8, signal to background greater than 35, and signal to noise greater than 24. Utilizing this assay, 313,816 compounds from the Molecular Libraries Small Molecule Repository were screened at 10 μM. We identified 7,583 compounds that showed greater than 22% CPE inhibition in the primary screen. The top 2,500 compounds were selected for confirmation screening and 409 compounds showed at least 50% inhibition of CPE and were considered active. We selected fifty-one compounds, based on potency, selectivity and chemical tractability, for further evaluation in dose response and secondary assays Several compounds had SI_50_ values greater than 3, while the most active compound displayed an SI_50_ value of 58.9.

**Conclusions:**

A robust automated luminescent-based high throughput screen that measures the inhibition of hRSV-induced cytopathic effect in HEp-2 cells for the rapid identification of potential inhibitors from large compound libraries has been developed, optimized and validated. The active compounds identified in the screen represent different classes of molecules, including aryl sulfonylpyrrolidines which have not been previously identified as having anti-hRSV activity.

## Background

Human respiratory syncytial viruses (hRSV) infect the lower respiratory tract and cause substantial morbidity and mortality annually [1]. hRSV disease is the most common cause of bronchiolitis and pneumonia among infants and children under one year of age [1]. Globally, there are approximately 3 million hospitalizations of children under five years of age and 200,000 deaths due to hRSV or its complications each year [2]. Even so, hRSV disease may occur at any age and severe disease can affect those individuals who have chronic obstructive pulmonary disease (COPD) and the immunocompromised [3]. In the United States, the economic impact of hRSV infections due to direct and indirect medical costs is greater than $650 million annually [4].

hRSV is a negative sense, single-stranded, non-segmented enveloped RNA virus of approximately 15 kb [5]. Ten viral genes encode 11 viral proteins that are transcribed from the viral RNA (vRNA). Three essential glycoproteins protrude from the membrane. The G-protein is thought to mediate attachment to long unbranched polysaccharides of the extracellular matrix composed of glycoaminoglycans (GAGs); however, virus that has a deletion of the G protein is able to replicate in tissue culture [6,7]. The fusion (F) protein is essential for fusion of the virus envelope with the cell membrane and entry of the virus into the cell cytoplasm through the interaction with protein RhoA. The small hydrophobic (SH) protein, a phosphoprotein, forms homopentamers suggesting it acts as a viroporin contributing to infection and replication [8,9]. The inner leaflet of the virion contains the mature (M) protein that coordinates the assembly of the virion. The viral core is composed of a nucleocapsid (N) protein that encapsidates the vRNA and binds to the L protein or RNA-dependent RNA polymerase (RdRp), phosphoprotein (P), and the transcription anti-terminator factor (M2-1) [10-12] to form the ribonucleoprotein (RNP) complex [13]. There are two non-structural proteins, NS1 and NS2 that may play a role in virus replication [14] and a regulatory protein (M2-2) [15].

Although hRSV was discovered more than 50 years ago, there is no FDA-approved vaccine. In 1966, a vaccine consisting of formalin-inactivated, parainfluenza virus and *Mycoplasma pneumoniae* caused severe bronchiolitis and pneumonia requiring hospitalization in 80% of vaccinated children upon hRSV challenge [16]. Tragically, two of the vaccinated infants succumbed to the symptoms of the disease [16,17]. Consequently, development of a vaccine is proceeding cautiously. The existing therapies for the acute infection are ribavirin [18,19] which has inconsistent clinical results and severe toxic liabilities, and the prophylactic humanized monoclonal antibody Synagis® (Palivizumab) that is limited to use in high risk pediatric patients [20]. Ribavirin, a nucleoside anti-metabolite prodrug also resembles GMP and can decrease cellular GTP pools due to the inhibition of the enzyme inosine monophosphate dehydrogenase (IMPDH) [21]. Nevertheless, this decrease does not completely account for the observed antiviral activity as inhibitory effects have been noted on RNA capping [22] and direct suppressive effect on the polymerase activity in the case of influenza viruses [23].

To date, screening efforts [24-26] have been used to discover lead candidate antiviral compounds against hRSV, which include inhibitors of IMPDH [27,28], viral fusion [29-32], the ribonucleoprotein (RNP) complex [33,34], virus attachment [35,36] and a nucleocapsid inhibitor [24]. However, many of these inhibitors did not advance into the clinic due to proper oral formulation for bio-availability [35] strategic reasons [37] and *in vivo* efficacy assessment that showed a reduction in pyrimidine pools rather than a decrease in viral load [38]. Hence, new drug candidates and treatment therapies are needed to combat hRSV. Toward this goal, we report herein on the development, optimization and validation of a 384-well cell-based assay that measures cytopathic effect (CPE) induced in HEp-2 cells by hRSV infection, using a luminescent-based detection system for signal endpoint. The validated assay was used to screen greater than 313,000 compounds from the Molecular Libraries Small Molecule Repository (MLSMR) and resulted in several new lead candidates.

## Results

### Optimization of media and cell density

Our initial efforts aimed to develop and optimize a high-throughput assay for efficient screening of small molecule libraries that measures the virus-induced cytopathic effect (CPE) in HEp-2 cells. During assay development, we optimized several parameters such as assay media (pH), plating density, assay DMSO tolerance, multiplicity of infection (MOI), and positive control drug concentration.

**Figure 1:**
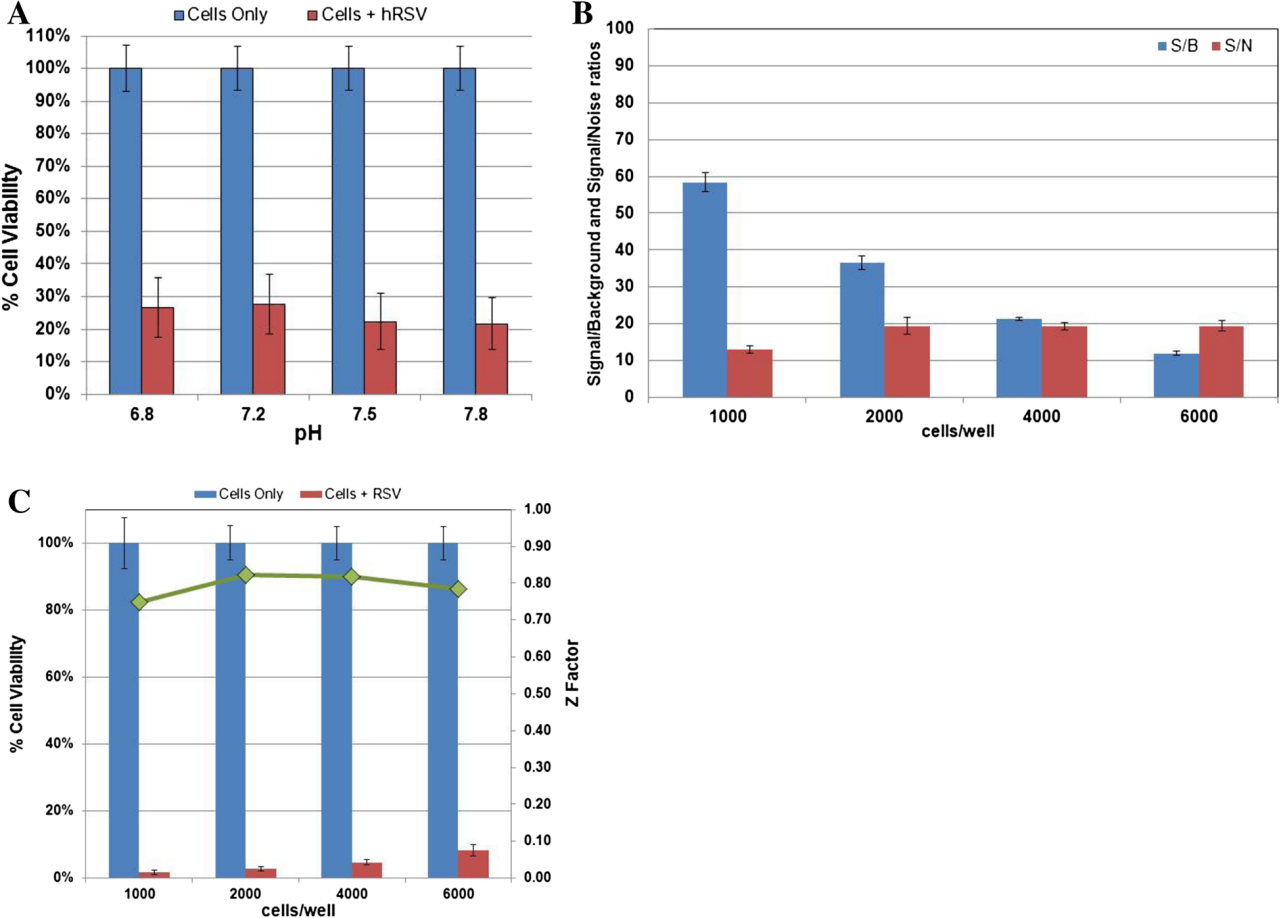
**Optimization of cell density. A)** HEp-2 cells were resuspended in DMEM/F12 medium at different pHs and plated into 384-well plates at 3000 cells per well. **B)** HEp-2 cells were plated into 384-well plates at the indicated cell number per well. The percent cell viability was used to calculate the signal-to-background and signal-to-noise ratios as per the Materials and Methods. **C)** HEp-2 cells were plated into 384-well plates at the indicated cell number per well. The percent cell viability was used to calculate Z factors as per the Materials and Methods. For all assays 100 TCID_50_ doses of RSV were added to the appropriate well which corresponds to an MOI of 0.2 infectious particles/cell. Percent cell viability was determined by the addition of Cell Titer Glo reagent (Promega). Error bars indicate the range from triplicate plates.

The reagent used to measure CPE was Cell Titer Glo Luminescent Cell Viability reagent. Cell Titer Glo generates a luminescent signal directly proportional to the amount of cellular ATP present which is proportional to the number of metabolically active cells. We began by determining the medium that would provide optimum virus infectivity in HEp-2 cells in a high throughput format (384-well format). After examining Opti-MEM, DMEM and DMEM/F12 supplemented with FBS concentrations ranging from 1 to 10%, we determined that complete DMEM/F12 with 2% FBS was the optimal assay medium (data not shown). In addition, we examined the pH of DMEM/F12 assay media. To determine optimum media pH, HEp-2 cells were resuspended in DMEM/F12 medium at pHs ranging from 6.8 to 7.8 and plated into 384-well plates at 3,000 cells per well. One half of the plates (n =192 wells) of each media pH were infected at an multiplicity of infection (MOI) of 0.2 plaque forming units (pfu) per cell and luminescence measured six days post-infection. There was 25 to 30% cell viability in the virus-infected cells at pH 6.8 and 7.2. Statistically, there was little difference between a media pH of 7.5 and 7.8 (20% cell viability in virus-infected cells); therefore, we chose a more biologically relevant pH of 7.5 as optimal (Figure 1A).

Optimal cell density is necessary to ensure that sufficient cells are present for adequate endpoint. A signal-to-background (S/B) ratio of greater than 5 and a signal-to-noise (S/N) ratio of greater than 10 is acceptable for most HT screening assays. Therefore, to determine the number of cells required to optimize S/B and S/N ratios cell density experiments were performed. We plated 1000, 2000, 4000, and 6000 HEp-2 cells per well in 384 well plates, infected the appropriate wells (n =192) with virus at an MOI of 0.2, incubated for six days, measured luminescence and determined percent cell viability. As shown in Figure 1B, 1000 cells per well had a S/B of 58.4 as compared to 12 for 6000 cells per well. The S/N values ranged from 13 for 1000 cells per well to approximately 19 for 2000, 4000 and 6000 cells per well. In addition to determining the S/B and S/N ratios we also determined the Z factor [39] of the plates with the various cell densities (Figure 1C). The Z factors for the plates containing 1000, 2000, 4000, and 6000 cells per well were 0.75, 0.82, 0.82 and 0.78, respectively (Figure 1C). We chose 2000 cells per well as optimal, since the S/B was 36.5, the S/N was 19 and the Z factor was 0.82 (Figure 1B and C).

### DMSO effect and virus multiplicity of infection

**Figure 2:**
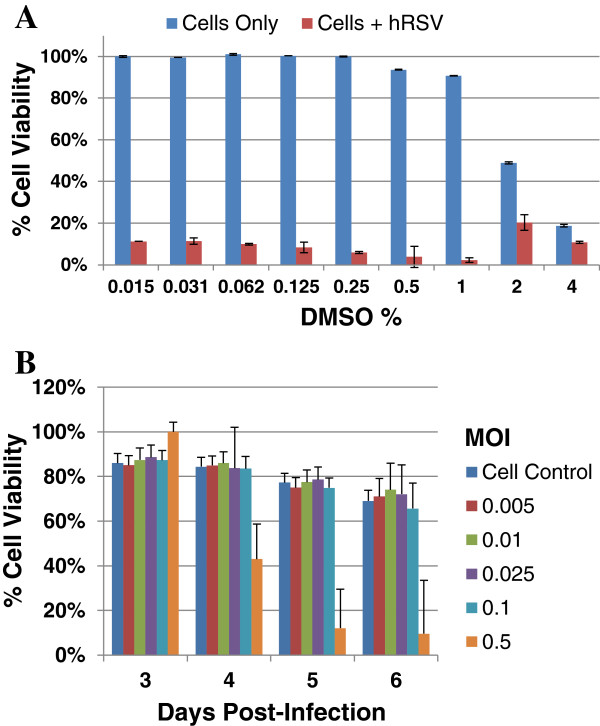
**DMSO effect on assay performance and hRSV Long strain MOI titration. A)** Serial 2-fold dilutions of DMSO in assay media, along with RSV Long strain [MOI = 0.2], were added to 2000 HEp-2 cells in 384 well plates. Six days post-infection, the cytopathic effect (CPE) of DMSO was assessed using Cell Titer Glo. **B)** Percent cell viability versus increasing MOIs on days 3 – 6 post-infection. CPE was assessed using Cell Titer Glo.

A critical parameter in assay performance is DMSO tolerance since it is used as a compound solvent. To determine the highest DMSO concentration that the HEp-2 cells could tolerate, we monitored the effect of serial 2-fold dilutions of DMSO (4% to 0.03%). Inhibition of cell growth was evaluated relative to uninfected cells and virus-infected cells treated with DMSO. The data indicates there is only a 10% reduction in HEp-2 cell viability with DMSO concentrations up to 1% (Figure 2A). Thereafter, cell viability declined substantially with increasing concentrations of DMSO as only 30% of the uninfected cells are viable at 3% DMSO. Moreover, DMSO concentrations of up to 2% did not inhibit virus-infectivity. However, we observed a steady decrease in the infected cell viability above 2% DMSO which parallels the DMSO toxicity curve and could be due to the combined result of toxicity and viral infection. Based on these findings, screening was performed at 0.5% DMSO final concentration.

To establish the signal dynamic range of the assay, we ascertained the optimal virus concentration that would give the largest difference between cells alone (the cell control) and cells infected with virus (the virus control). Dilutions of hRSV (MOI = 0.005, 0.01, 0.025, 0.1 and 0.5) were added to HEp-2 cells (2000 cells/well) and luminescence was measured on days 3 to 6 post infection. MOIs ranging from 0.005 – 0.1 caused CPE in only 20% of cells (80% cell viability) when determined 6 days post-infection (Figure 2B). However, HEp-2 cells infected with virus at an MOI of 0.5 showed 20% cell viability. Therefore, we determined the optimal concentration of the virus relative to the cell control was with an MOI of 0.5 on day 6 p.i.

### Control drug concentration

**Figure 3:**
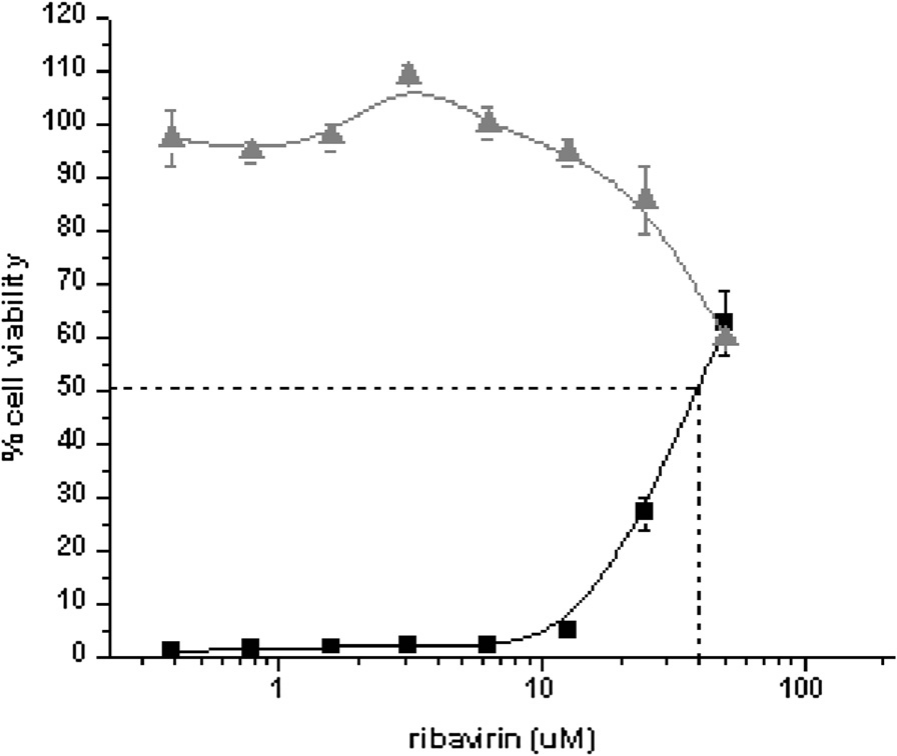
**Dose response of ribavirin.** HEp-2 cells were plated in 384-well black tissue culture plates at 2,000 cells per well. Ribavirin or carrier control (DMSO) were diluted to 6x in Complete DMEM/F12 and serially diluted 1:2 resulting in an 8 point dose response dilution series. (final plate well concentration ranging from 50 uM to 0.39 uM and a final DMSO concentration of 0.5%). For dose response screening, cells were infected with a 1:500 dilution of virus (viral MOI = 0.5). The assay plates were incubated for six days at 37°C, 5% CO_2_ and 90% relative humidity and the inhibitory effects of the drug were assessed using Cell Titer Glo. The EC_50_ value (squares) was performed in triplicate while the IC_50_ value (triangles) was performed in duplicate.

The only FDA approved antiviral drug for acute hRSV infection is ribavirin [18,19]. We used the CPE based assay to evaluate the metabolically active form of ribavirin [40] in dose response experiments for EC_50_ (efficacy) and CC_50_ (cytotoxicity). As is shown in Figure 3 we determined an EC_50_ value of approximately 40 μM as compared to 41 μM from previously reported EC_50_ values [41] and an CC_50_ value of approximately 75 μM. Given the narrow window between ribavirin efficacy and toxicity, we chose 35 μM dose of ribavirin as the positive control drug concentration.

### Z-factor analysis and assay performance

As a final parameter to validate the robustness of our assay, adapt to the HTS platform and assess the quality of this assay, we performed Z-factor and coefficient of variation (CV) analyses by running three separate plates on three days for a total of 9 plates. We plated 2000 HEp-2 cells per well of a 384-well plate and infected half the plates with hRSV at an MOI of 0.5. We obtained Z factors greater than 0.8 (0.82 ± 0.02), however, the CV values for the virus-infected wells averaged above 20% (24.8 ± 0.01%). We attributed these results to the instability of hRSV. For example, during assay development and early validation, virus stocks retained infectivity for varying lengths of time from one to six weeks [42]. The high CV value reflected inconsistency of virus infectivity. To overcome this problem, we produced frozen hRSV-infected HEp-2 cells and mixed them at a ratio of 1:100 with uninfected Hep-2 cells. Previously, we showed a Pearson's correlation of 0.84 of duplicate EC_50_ values derived from 92 compounds using frozen hRSV-infected HEp-2 cells versus high-titer infectious virus. These data suggested we could use the frozen infected cells as a source of infectious pathogen [42].

**Figure 4:**
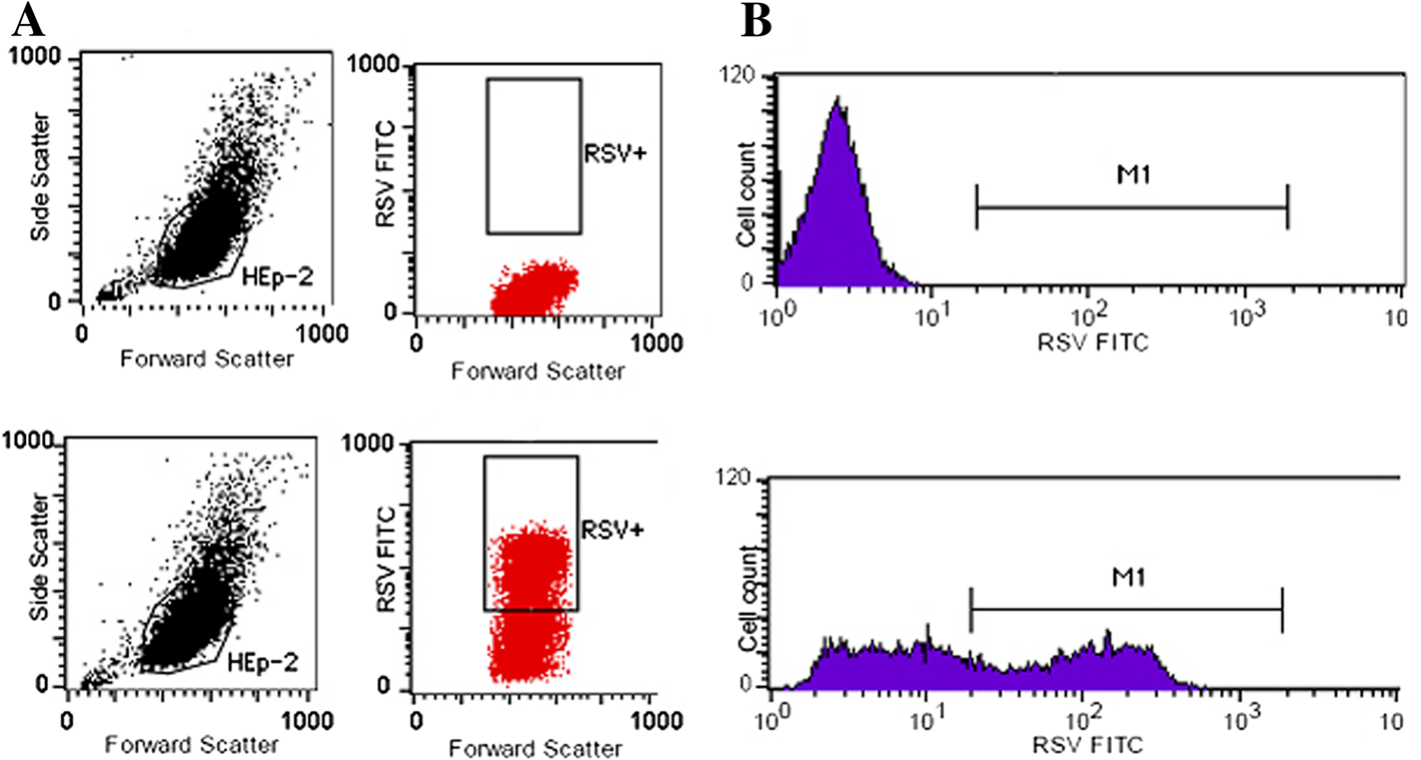
**FACS analysis of frozen RSV-infected HEp-2 cells. A)** Flow cytometry evaluation of infectivity. Representative dot plots of forward scatter versus fluorescence of HEp-2 cells infected in vitro with RSV strain Long. A minimum of 10000 cells were acquired for each dot plot. The percentage of positive cells in the FACS analysis represents the percentage of RSV positive cells over the live cell population. This was calculated applying a forward and side-scatter gate to eliminate dead cells and debris from the analysis. **B)** Intracellular staining in HEP-2 cells infected with RSV strain Long. Histograms of fluorescence of cells stained intracellularly with mouse monoclonal [4 clone blend] to RSV. Controls included unstained cells and cells stained with antibody and uninfected cells stained with antibody.

To ascertain the percentage of HEp-2 cells that were infected with hRSV in our assay we examined them by flow cytometry. hRSV-infected HEp-2 cells were stained intracellularly with mouse monoclonals to hRSV. We determined that 41% of the cells or approximately 820 of 2000 cells per 384 well were infected with hRSV (Figure 4). Hence, hRSV- infected cells were utilized in the HT assay to screen the MLSMR.

### Compound screen results

**Figure 5:**
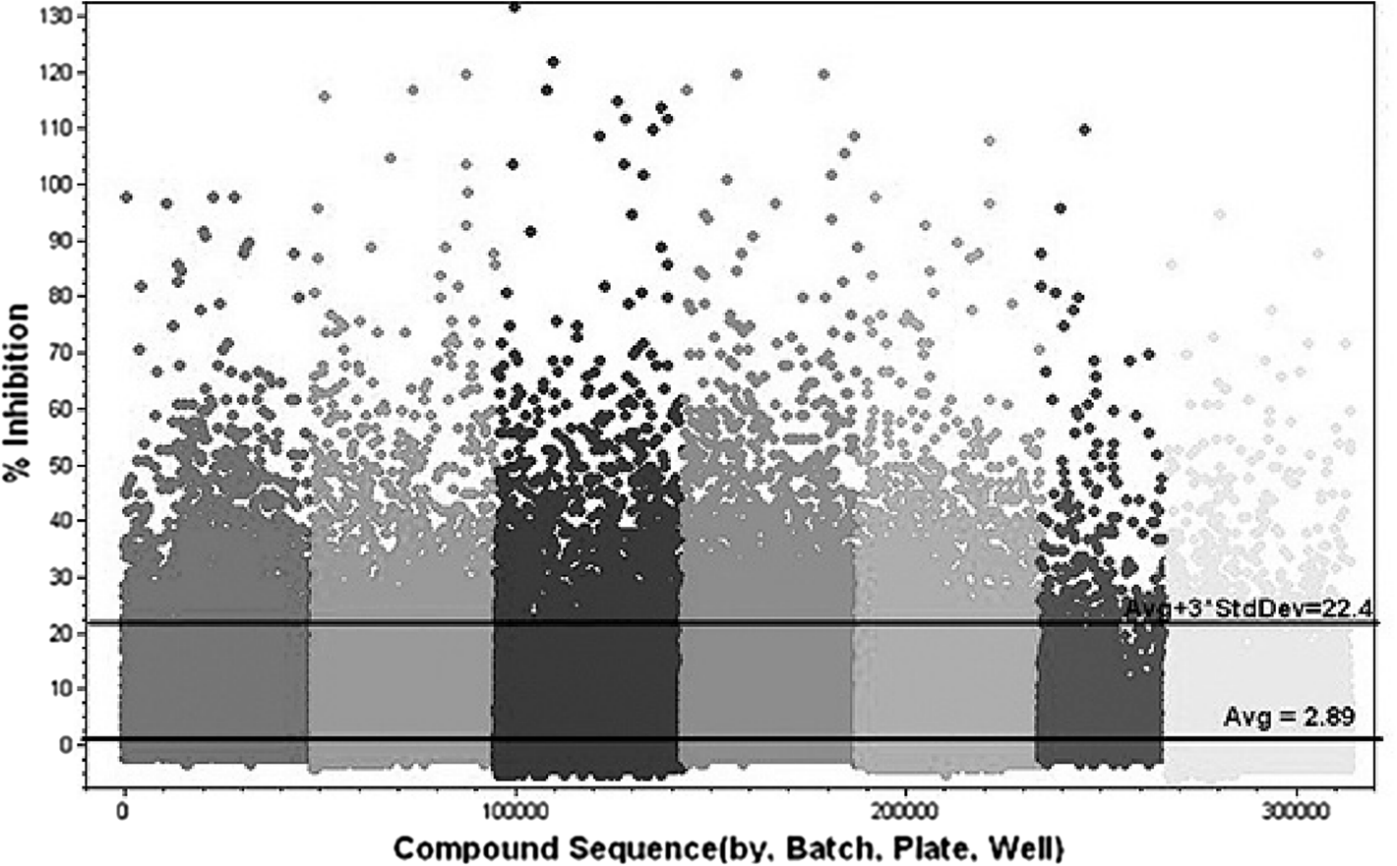
**HTS of MLSMR library.** High through-put screen of 313,816 compounds in single dose from the NIH MLSMR library against RSV. Compounds from the library, along with the respiratory syncytial virus, strain Long, frozen-infected HEp-2 cells were added to uninfected HEp-2 cells per well in 384-well plates. Inhibitory effects were assessed after 6 days, as described in Materials and Methods. Reference lines were calculated from the percent (%) inhibition.

A total of 313,816 compounds from the MLSMR were screened in single dose at 10 μM compound concentration against hRSV strain Long HEp-2 infected cells. The average Z-factors were 0.8 ± 0.08 with an average signal to background of 37.6 ± 6.2 for the single dose screening campaign. The mean inhibition of the compound wells was 2.9%. Inhibition values of greater than 22.4% were outside the calculated noise of the assay defined by the average percent inhibition plus three times the standard deviation (3σ ) (Figure 5). We identified 7,583 compounds that showed greater than 22% CPE inhibition in the primary cell-based HT screen for a “hit” rate of 2.4%.

**Figure 6:**
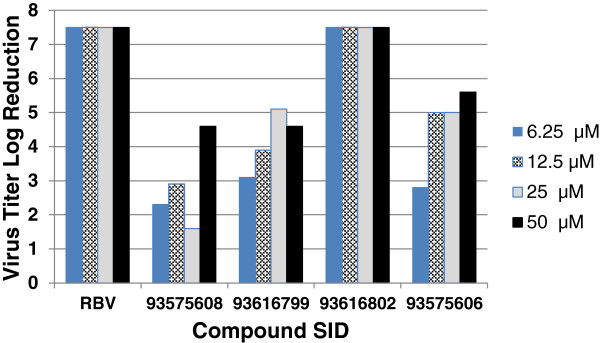
**Titration of progeny viruses.** Titer of progeny viruses produced from the cell was measured by TCID50 assay in 384-well plate format with 4 wells per dilution of virus. Ten (10) μL of 10-fold serial dilutions of progeny virus containing medium from respective samples (drug treated or untreated) were used to infect fresh Hep-2 cells in a 384-well format. After 6 days incubation the inhibitory effects of the compound and drug were assessed using Cell Titer Glo. A well showing a luminescence signal less than the mean of the non-infected control signal minus five times the standard deviation of the control was regarded as positive for infection.

The top 2,465 compounds were selected for confirmation screening based on the criteria of greater than or equal to 50% CPE inhibition. Of the compounds tested in the confirmatory screen, 409 compounds showed at least 50% inhibition of CPE and were considered active. We selected fifty-one compounds, based on potency, selectivity and chemical tractability, for further evaluation in dose response and measured the difference in viral titer between non-treated and treated cells in a titer reduction assay. The titer reduction assay involves challenging HEp-2 cells with hRSV at a high MOI in the presence of compound. The progeny virus was serially diluted 10-fold and the suspension transferred to fresh HEp-2 cells in 384-well plates. The progeny viral titer was calculated using the TCID_50_ method [43]. Of the 51 compounds analyzed by TCID50 analysis, all showed more than 10-fold reduction in the progeny titer (<-1 of log reduction) for at least one concentration (Figure 6).

**Figure 7:**
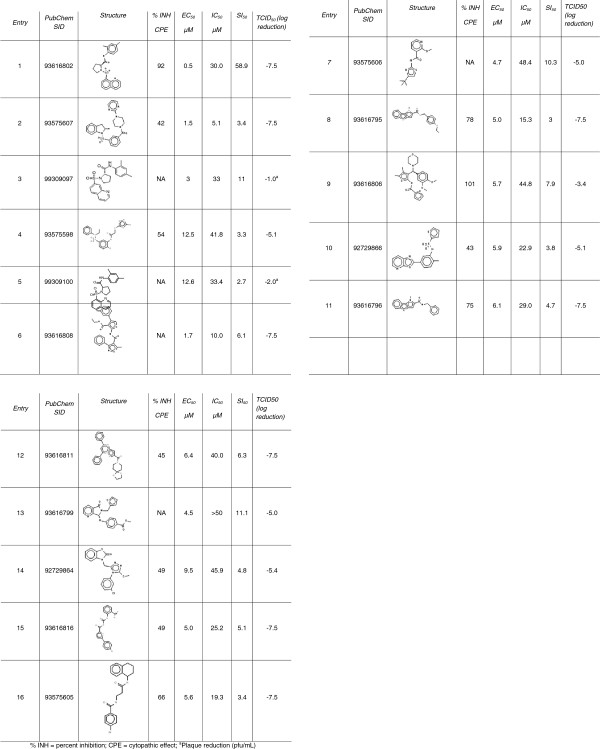
Structures and inhibitory activities of selected compounds (μM).

Four general chemotypes (or chemical families) emerged from the TCID50 analysis. They are represented in Figure 7. The *N*-alkylsulfonamides are represented by entries 1-5 and the bis-arylamides and isosteric bis-arylsulfonamides by entries 6-12. The other two clusters are both mixed aryl/heterocycles, however one chemotype is separated by a single linker atom (entries 13 and 14), while the other is separated by an amide-based linker chain (entries 15 and 16). The structures and inhibitory activities of selected compounds in Figure 7 are sorted by cluster and by increasing EC_50_ values within each chemical family.

### Inhibition of progeny virus production - hRSV plaque assay

**Figure 8:**
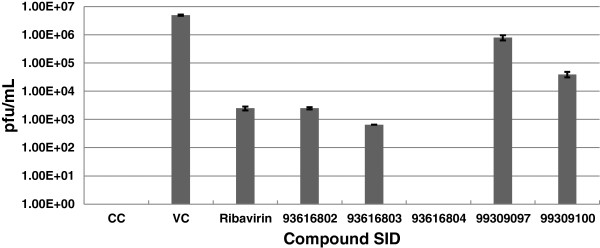
**Plaque reduction assay in HEp-2 cells.** RSV strain Long was assayed for its sensitivity to five compounds. Supernatants were harvested from test (25 μM) or control compound (35 μM) treated RSV-infected HEp-2 cells at a multiplicity of infection (MOI) of 0.1. Serial dilutions of the supernatants were added to monolayers of HEp-2 cells in 24-well plates. Cells were fixed after 6 days and stained with neutral red. Plaque reduction assays were performed in triplicate per compound. CC, uninfected cell control; VC, untreated virus-infected control. The concentration of DMSO in each assay well, including all control wells was 0.5%.

A plaque assay was used to confirm the antiviral compound effect of a selected set of compounds possessing sulfonamide and carboxamide functions. These compounds were evaluated for their ability to reduce the amount of infectious virus produced in cell culture. HEp-2 cells were infected with hRSV in the presence of 25 μM test compound. Media supernatants were removed and 10-fold serial-diluted onto a confluent field of uninfected, untreated HEp-2 cells, bound, washed, and overlaid with agarose. After six days, plaques were fixed, stained, and counted to determine the amount of infectious virus in the original, drugged supernatants (Figure 8). Compounds with SIDs 93616802 and 93616803 exhibited a 3-log and 4-log reduction in virus titer, respectively, while SID 99309097 and SID 99309100 reduced titers 1 to 2 fold. Most interestingly, SID 93616804 exhibited a 6.5-log reduction from the control in pfu/mL.

## Discussion

A whole cell HT assay for the rapid identification of potential inhibitors of hRSV from large compound libraries has been developed, optimized and validated. The assay is sensitive and robust, with Z factors equal to 0.8, signal to background greater than 35, and signal to noise greater than 24. Our cell based assay utilizes the luminescence signal generated by the amount of ATP present in the samples to measure the inhibition of virus-induced cytopathic effects on cells [42]. This CPE-based assay has an advantage over reporter-based assays as it can be used to identify inhibitors that target multiple steps involved in viral replication and to screen for cytoxicity.

Using this HT assay we screened greater than 313,000 small molecules from the MLSMR at 10 μM for inhibition of hRSV *in vitro*. We identified more than 7,500 compounds that showed at least 22% inhibition of CPE for a hit rate of 2.4%. We had a confirmation hit rate of 16.5% as 409 compounds out of more than 2,400 compounds screened in dose response/cytotoxicity assays met our criteria of activity: the efficacy, EC_50_ value of less than 15 μM and with toxicity to efficacy, SI_50_ of greater than 3.

The compounds presented in Figure 7 represent a selected set of compounds possessing sulfonamide and carboxamide functions and were chosen on the basis of several considerations including the fact that these compounds displayed at least a 3-log reduction in virus as determined by preliminary TCID_50_ analysis. Furthermore, three compounds displayed at least a three-fold reduction from the control (pfu/mL) in a plaque reduction assay.

To begin to probe the mechanism of action of the sulfonylpyrrolidines, the window of inhibitory activity in the cell-based assay was refined. Potency of compounds over time following infection was examined to ascertain early (entry) or late (replication) antiviral activity in the virus life cycle [44]. In the time of addition study, HEp-2 cells were infected with hRSV strain Long at an MOI of 0.1 at time point 0 and test compounds or ribavirin were added to plates one hour before infection to 24 hours after infection. Six days later, CPE was assessed using Cell-Titer Glo as an endpoint reagent. An *N*-alkylsulfonamide compound with SID 99309100 demonstrated a decrease in efficacy when added at each time point from 0-5 hours p.i. This profile could be due to the inhibition of one or more early virus life cycle steps (entry, post-entry, or early-stage infection processes suggesting this chemotype inhibits early infection events, characterized by viral attachment, uptake, fusion or initial transcription.

There are several promising candidates in various stages of clinical development that could meet the need for the Paramyxovirus antiviral market; however, drug resistance may lead to a need for additional compounds in the pipeline. Studies in the laboratory reveal that resistant viruses arise after 15-20 days in culture in the presence of the palivizumab. Sequence analysis reveals that a single amino acid change in the fusion protein produces a virus that is as fit as the wild type [45-47]. While there are no reports of Synagis resistant viruses circulating in the population, a palivizumab-resistant hRSV infection from an infant has been documented [48]. Two mutants in the F protein encompassing amino acids 262-276 conferred resistance to palivizumab. Thus, there has been promising data to suggest that hRSV polymerase and fusion proteins are pharmacological targets that merit the development of multiple drugs to abort infection and circumvent drug resistance.

## Conclusions

The aim of this study was to develop, validate and optimize a cell-based HT assay for identification of new hRSV antiviral lead compounds from the MLSMR. Primary and secondary screening of the library has led to the discovery of several compounds with SI values of greater than 3. Dose response and cytotoxicity assays indicate that at least one of the scaffolds met our activity criteria for further SAR evaluation: an efficacy EC_50_ value of < 15 μM and toxicity to efficacy SI of >3. This scaffold has not been previously identified as having anti-viral activity. Subsequently, several analogues have been synthesized for additional testing, analyzed in-depth to evaluate SAR and contribute to probe optimization efforts. The results of such an extended study have been disclosed in a separate publication [44].

## Methods

### Cell growth conditions and media

HEp-2 cells (ATCC CCL-23, American Tissue Culture Type) were maintained as adherent cell lines in Opti-MEM1® (Invitrogen) with 2 mM L-glutamine and 10% fetal bovine serum (FBS) at 37°C in a humidified 5% CO_2_ atmosphere. Cells were passaged as needed and harvested from flasks using 0.25% trypsin-EDTA.

### Assay media

Preparation of Complete DMEM/F12 was as follows: 50 mL Pen/Strep/Glutamine (Gibco) was added to four liters of room temperature DMEM/F12 (Sigma) and the pH adjusted to 7.5 using 1 N NaOH. The medium was sterile filtered (0.2 μm) and 10 mL of HI-FBS (Gibco) was added per 500 mL of media.

### hRSV culture

Human respiratory syncytial virus (hRSV) strain Long (ATCC VR-26) was used for assays. Virus was serially diluted and a dilution of 1:10 was used to amplify the seed stock. Briefly, a TCID_50_ format of 10-fold serial dilutions (from 10^-1^ to 10^-7^) was used to dilute the virus. HEp-2 cells grown in a 384-well plate were infected with hRSV. Plates were incubated at 37°C, 5% CO_2_, and 90% relative humidity for four days. Supernatant from the 384-well plate’s highest viral dilution was used to infect a single well of HEp-2 cells in a 6-well plate format containing approximately 2.4 × 10^6^ cells/well. A two mL volume of Complete-Opti-MEM1 (C-Opt1) (Gibco) containing 10% FBS per well was removed and replaced with 100 μL of C-Opt1. Virus culture supernatant from the 384-well TCID_50_ was added to 100 μl C-Opt1 and incubated at 37°C, 5% CO_2_, and 90% relative humidity for 1.5 h rotating every 30 min to facilitate infection. The media was removed and replaced with 2 mL of C-Opt1 and incubated at 37°C, 5% CO_2_, and 90% relative humidity. After 72 h, the supernatant was removed and the cell debris pelleted by centrifugation at 300 *× *g, 5 min, at 18°C. One T-175, containing 4.78 × 10^6^ HEp-2 cells was incubated overnight and used to amplify the virus. After 18 h, media was removed, cells were washed with 10 mL Complete-DMEM/F12 (2% FBS, 1.25% P/S/G, pH 7.5) and replenished with 4 mL C-DMEM/F12. A 100 μL sample of clarified hRSV was added to a T-175 and incubated for 1.5 h at 37°C, 5% CO_2_, and 90% relative humidity. The media was removed and replenished with 25 mL of C-DMEM/F12, and incubated at 37°C, 5% CO_2_, and 90% relative humidity for 48 h. The media was transferred to a 50 mL conical tube and cell debris pelleted at 300 × g, 5 min, at 18°C. Trehalose and FBS were added to a final concentration of 10% (v/v) each for preservation [49] and the supernatant was aliquoted (1 mL per tube), fast frozen in 100% EtOH/dry ice and stored at –150°C. Virus stocks titers were quantified in HEp-2 cells using an agarose overlay plaque method. The titer of the virus was 1.0 × 10^7^ pfu/mL.

### Infectious material: Frozen infected virus cell preparation

Preparation of the frozen hRSV-infected HEp-2 cells has been previously described [42]. Briefly, a T-225 flask containing 3.0 × 10^8^ HEp-2 cells in 30 mL Complete DMEM/F12, pH 7.5, was grown to 95% confluence. Two mL hRSV (strain Long) containing 1 × 10^7^ pfu/mL was added to the flask and incubated for 18 – 20 h at 37°C, 5% C0_2_, 90% relative humidity. After incubation, the medium was aspirated and the cells washed with 10 mL PBS without Mg^2+^ or Ca^2+^. Cells were harvested from flasks using 0.25% trypsin-EDTA. Cells were centrifuged at 300 × *g* for 10 min and re-suspended in 95% FBS, 5% DMSO at a concentration of 2 × 10^6^ cells/mL. The cells were determined to be at least 99% viable. The cells were aliquoted in 1 mL aliquots, rate frozen at –1°C/min to -80°C and stored at –150°C. Viability was also evaluated when thawed and determined to be at least 98.5%. We confirmed the percentile of infected cells in two ways; immunostaining and cell counting using FACS and a limited dilution methodology.

### FACS analysis of frozen infected cells

Frozen hRSV-infected and un-infected HEp-2 cells (2 × 10^6^ cells) were centrifuged at 300 × g for 5 min and the supernatant removed. Cell pellets (uninfected and hRSV-infected) were fixed in 1 mL of 4% paraformaldehyde for 15 min on ice. Cells were washed twice in 1 mL staining buffer (1X - Dulbecco's Phosphate Buffered Saline Solution [DPBS], 2% FCS) centrifuging at 300 × g for 5 min between washes. Cells were resuspended in 1 mL of staining buffer and 2 × 10^5^ cells were aliquoted into 12 × 75 mm tissue culture tubes. Dilutions (1:5000, 1:2000, 1:1000, 1:500) of mouse monoclonal [4 clone blend] to hRSV(Abcam) was added to the cells, incubated for 30 min on ice and washed twice in 3.5 mL staining buffer. Secondary antibody (goat anti-mouse IgG/M conjugated to FITC (BD Pharmingen) was added, incubated for 30 min on ice and washed twice with 3.5 mL DPBS. Cells were resuspended in 0.4 mL DPBS and analyzed on a FACSCalibur flow cytometer. Controls included unstained cells, cells stained with either the primary or secondary antibody and uninfected cells stained with both antibody reagents.

### Compound libraries and controls

The positive control drug for this assay, ribavirin (MP Biomedicals, Solon, OH) was solubilized in DMSO, diluted and added to the assay plates as described for test compounds. Final concentration for ribavirin was 35 μM. All wells contained 0.5% DMSO.

The MLSMR is a library of biologically relevant small organic molecules that has been utilized for HTS as part of the NIH Roadmap initiative, the Molecular Libraries Production Center Network (MLPCN). This library has been updated and expanded since the initiation of the program in 2005. Compounds were solubilized at 10 mM in DMSO and all compounds were diluted in assay media for a final concentration of 10 μM in the screen. The concentration of DMSO in each assay well, including all control wells was 0.5%.

### Compound preparation

For single dose screening in a 384 well plate format, compounds or carrier control (DMSO) were diluted to 6× in Complete DMEM/F12 using a Biomek FX and 5 μL was transferred to the assay plate. Cells were added to the plate in 25 uL of media using a Thermo/Matrix Wellmate. Final plate well concentration was 10 μM compound, 2,000 cells, and 0.5% DMSO in a total volume of 30 uL.

For dose response screening in a 384 well plate format, compounds or carrier control (DMSO) were diluted to 6× in Complete DMEM/F12 using a Biomek FX and 5 μL was dispensed to assay plates (3% DMSO). Test compounds were serially diluted in a plate to plate matrix or “stacked plate” matrix. All 320 compounds in a source plate were diluted together resulting in a 10 point dose response dilution series proceeding vertically through a stack of plates with the high dose plate on top and the low dose plate on the bottom (final plate well concentration ranging from 50 μM to 0.097 μM and a final DMSO concentration of 0.5%).

### Assay setup

Compounds or carrier control (DMSO) were diluted to 6x in C-DMEM/F12 and 5 μL was dispensed to 384-well assay plates (3% DMSO or 60 μM compound in 3% DMSO). Twenty five μL of uninfected HEp-2 cells were plated in the cell control wells. Frozen hRSV-infected cells were combined with uninfected HEp-2 cells at a 1:100 ratio. Twenty five μL of the cell mixture was added to the virus control and compound wells. All cell plating was conducted using a Matrix WellMate and cells were maintained at room temperature with stirring during the plating process. The assay plates were incubated for six days at 37°C, 5% CO_2_ and 90% relative humidity.

### Endpoint read

Following the six day incubation period, the assay plates were equilibrated to room temperature for 30 min. An equal volume (30 μL) of Cell Titer-Glo reagent (Promega Inc.) was added to each well using a WellMate (Matrix, Hudson, NH) and the plates were incubated for an additional 10 min at room temperature. At the end of the incubation, luminescence was measured using a multi-label reader (Envision, PerkinElmer, Wellesley, MA) with an integration time of 0.1 s.

### Data analysis

HTS data were analyzed using ActivityBase software (IDBS, Inc., Guildford, UK). Antiviral activity is described as percent CPE inhibition = 100*((luminescence compound well minus median luminescence virus control)/ (median luminescence cell control minus median luminescence virus control)). Percent viability = 100 * luminescence compound well/median luminescence cell control. An active compound, or “hit,” was defined as a compound that exhibited a % CPE inhibition of >22% without compromising cell viability. Two dose-response curves were calculated for each substance. One assessed % CPE inhibition at each dose (EC50); the other assessed cytotoxicity at each dose (CC50). EC50 values (for % CPE inhibition) and CC50 values were calculated using the 4-parameter Levenburg-Marquardt algorithm with parameter A locked at 0 and parameter B locked at 100. Standard deviation, normalized chi^2^, and Hill slope were used to evaluate the curves. Values were not extrapolated beyond the tested range of concentrations. The selective index (SI) was calculated as SI = CC_50_/EC_50_. The criteria for determining compound activity are based on its SI. Compounds with an SI value of >3 were defined as active, whereas compounds that exhibited an SI value less than 3 were defined as inactive.

Thirty-two control wells containing cells only and 24 wells containing cells and virus were included on each assay plate and used to calculate Z factors for each plate and to normalize the data on a per plate basis. Eight ribavirin positive control wells were included on each plate for quality control purposes but were not used in Z calculations.

The Z factor values were calculated from 1 minus (3*standard deviation of cell control plus 3* standard deviation of the virus control / [mean cell control signal minus mean virus control signal [39]. The signal-to-background (S/B) was calculated from mean cell control signal divided by the mean virus control signal. The signal-to-noise (S/N) was calculated from mean cell control signal minus mean virus control signal divided by the standard deviation of the cell control signal minus the standard deviation of the virus control signal [39].

### Titration of progeny viruses

Titer of progeny viruses produced from the cell was measured by TCID50 assay in 384-well plate format with 4 wells per dilution of virus. Ten μL of 10-fold serial dilutions of progeny virus containing medium from respective samples (drug treated or untreated) were used to infect fresh Hep-2 cells in a 384-well format. The cell plates were incubated at 37°C, 5% CO_2_, and high humidity for an additional 6 days. The Cell Titer Glo assay was used to determine viability of the cells. A well showing a luminescence signal less than the mean of the non-infected control signal minus five times the standard deviation of the control was regarded as positive for infection.

### Plaque assays - compound preparation

Compounds or carrier control (DMSO) were diluted in Complete DMEM/F12 and 2 mL per well was dispensed to 6-well assay plates (final plate well concentration was 25 μM, ribavirin; 35 μM and final DMSO concentration 0.5%).

### Preparation of HEp-2 cells and plaque assay setup

HEp-2 cells were harvested and resuspended to 500,000 cells per mL in Complete DMEM/F12 and seeded in 6 well tissue culture plates at 1,000,000 cells per well in 2 mL Complete Optimem1 and incubated 24 hours at 37°C, 5% CO_2_, 90% relative humidity. The media was aspirated from the wells, 0.5 mL hRSV Long strain (MOI of 0.1) diluted using C-DMEM/F12 was added and the plates incubated at 37°C, 5% CO_2_, rotating every 20 min. to facilitate infection. After 2 hours, the virus supernatant was aspirated and each well was washed with 3 mL of 1X PBS. Compounds were diluted in C-DMEM/F12 media to give a final concentration of 25 μM, added to assay plates and incubated at 37°C, 5% CO_2_ and 90% relative humidity. After 48 h, the supernatant (hRSV/compound/media; 1.6 mL) was removed, flash frozen on dry ice, and stored at –80°C.

HEp-2 cells in Complete Optimem1were seeded in 24 well tissue culture plates at 400,000 cells per well in 0.5 mL and incubated 24 h at 37°C, 5% CO_2_. The supernatant (hRSV/compound/ media) was removed from -80°C and thawed on ice. The supernatants were serially diluted in Complete DMEM/F12 media (10^-1^ to 10^-4^). The media was aspirated from the 24 well plates, 0.2 mL of each supernatant dilution was added to each well and the plates incubated at 37°C, 5% CO_2_, rotating every 20 min. to facilitate infection. After 2 h, each well was washed one time with 1X PBS followed by the addition of 0.5 mL 1% Avicel per well. The assay plates were incubated for six days at 37°C, 5% CO_2_ and 90% relative humidity.

### Staining of virus plaques

Following the six day incubation period, the Avicel overlay was aspirated, washed with 0.5 mL of 1X PBS, and fixed with 0.5 mL of 4% paraformaldehyde per well. The assay plates were incubated at 4°C for 24 h. The paraformaldehyde was aspirated, each well washed with 1 mL deionized water, and stained with 1 mL of 0.05% neutral red with periodic shaking for 10 minutes at room temperature. The neutral red was aspirated and the plates briefly inverted without lids on paper towels for drying.

## Competing interests

The authors declare that they have no competing interests.

## Authors’ contribution

DHC, BPM, LR, FJ, and CM carried out most of the primary and secondary screening and contributed to the interpretation of the results. MIS and LR analyzed the HTS data, uploaded to PubChem and contributed to the interpretation of the results. JEG designed analogs of the hit and evaluated data for the iterative SAR effort. DSM synthesized analogs for SAR exploration. LW contributed to the interpretation of the results and writing the manuscript. CBJ contributed to the early stages of assay development, interpretation of the results and writing the manuscript, WES contributed to the development and optimization of the primary screen, contributed to the interpretation of the results, drafted and finalized the manuscript. All authors read and approved the final manuscript.
